# Selenium supplementation in individuals with newly diagnosed Graves’ hyperthyroidism: a double-blind, multi-centre RCT

**DOI:** 10.1530/ETJ-25-0264

**Published:** 2026-01-06

**Authors:** Per K Cramon, Kristian H Winther, Victor B Boesen, Camilla B Larsen, Jakob B Bjorner, Selma F Nordqvist, Julie L Forman, Anne B Juul, Pernille Bach-Mortensen, Nils Knudsen, Runa L Nolsøe, Tina Vilsbøll, Alin Andries, Jeppe Gram, Birte Nygaard, Kamil Demircan, Thilo S Chillon, Lutz Schomburg, Laszlo Hegedüs, Steen J Bonnema, Ulla Feldt-Rasmussen, Åse K Rasmussen, Torquil Watt

**Affiliations:** ^1^ Department of Clinical Physiology and Nuclear Medicine, Copenhagen University Hospital – Rigshospitalet, Copenhagen, Denmark; ^2^ Department of Endocrinology, Odense University Hospital, Odense, Denmark; ^3^ Department of Nephrology and Endocrinology, Copenhagen University Hospital – Rigshospitalet, Copenhagen, Denmark; ^4^ IQVIA, Copenhagen, Denmark; ^5^ Department of Public Health, Section of Biostatistics, University of Copenhagen, Copenhagen, Denmark; ^6^ Department of Endocrinology, Copenhagen University Hospital – Amager and Hvidovre, Hvidovre, Denmark; ^7^ Department of Endocrinology, Copenhagen University Hospital – Bispebjerg and Frederiksberg, Copenhagen, Denmark; ^8^ Copenhagen University Hospital – North Zealand, Hillerød, Denmark; ^9^ Clinical and Translational Research, Steno Diabetes Center Copenhagen, Herlev, Denmark; ^10^ Department of Clinical Medicine, Faculty of Health and Medical Sciences, University of Copenhagen, Copenhagen, Denmark; ^11^ Department of Endocrinology, University Hospital of Southern Denmark, Esbjerg, Denmark; ^12^ Department of Endocrinology, Copenhagen University Hospital – Herlev and Gentofte, Herlev, Denmark; ^13^ Institute for Experimental Endocrinology, Charité-Universitätsmedizin Berlin, Berlin, Germany; ^14^ Medical and Science, Novo Nordisk, Søborg, Denmark

**Keywords:** Graves’ disease, Graves’ hyperthyroidism, selenium, quality of life, ThyPRO, pragmatic trial

## Abstract

**Purpose:**

We examined the effect of selenium vs placebo on remission rate and quality of life (QoL) in the Graves’ selenium supplementation (GRASS) trial (ID: NCT01611896).

**Methods:**

Double-blinded, placebo-controlled, multi-centre trial in individuals with newly diagnosed Graves’ hyperthyroidism randomised to daily supplementation with 200 μg selenium or placebo tablets during 24–30 months, depending on the timing of antithyroid drug (ATD) withdrawal. The primary outcome was the proportion of participants with non-remission, defined as receiving ATD or remaining hyperthyroid (thyroid stimulating hormone <0.1 mIU/L) during the last 12 months of the intervention period or referral to ablative therapy (radioactive iodine or surgery). QoL was serially assessed by the thyroid-related patient-reported outcome ThyPRO and compared with previously collected norm data.

**Results:**

Between Dec 7th 2012 and Dec 3rd 2018, 430 participants with Graves’ hyperthyroidism were recruited. Non-remission was observed in 114 (53.3%) participants in the placebo group and 118 (54.6%) in the selenium group (OR = 1.0 (95% CI: 0.7–1.5); *P* = 0.98). There was no beneficial effect of selenium, as compared with placebo, on any ThyPRO scale. The participants’ QoL at the end of the study was comparable to that of the general population sample. Thyrotropin receptor antibody levels were similar in the groups at the 18-month and end-of-study follow-up visits.

**Conclusion:**

In individuals with newly diagnosed Graves’ hyperthyroidism, daily supplementation with selenium did not have any effects compared with placebo as add-on to standard antithyroid drugs. The GRASS trial findings do not support the use of selenium supplementation in Graves’ hyperthyroidism.

## Introduction

Graves’ hyperthyroidism is an autoimmune disease caused by complex interactions between genetic and environmental factors. The disease is characterised by unregulated stimulation of the thyroid stimulating hormone (TSH) receptor by TSH receptor antibodies (TRAb), leading to thyrocyte proliferation and thyroid hyperfunction (1). In 40–50% of individuals with Graves’ hyperthyroidism, the disease may go into remission within approximately 1–2 years, reflected by undetectable TRAb levels and no need for antithyroid medication. Factors associated with persistent disease include an enlarged thyroid gland and high levels of thyroid hormones and TRAb at diagnosis (1). It is well established that individuals with Graves’ hyperthyroidism have short- and long-term quality of life (QoL) impairments, compared with data from the general population (2, 3). Symptoms and QoL impairment result from hyperthyroidism and its treatment but may also be a consequence of the underlying autoimmunity (1).

Selenium is a metalloid that is essential for the function of a number of enzymes in the body, including the iodothyronine deiodinases (4). The thyroid expresses a number of selenoproteins, including iodothyronine deiodinase type 1 and 2, and the thyroid gland has a higher concentration of selenium than most other organs. Furthermore, thyrocytes express selenoproteins with antioxidant effects (e.g. glutathione peroxidases and thioredoxin reductases) that protect against oxidative stress and thereby lower the activation of inflammatory signalling. Thyrocytes also express selenoproteins that downregulate the transcription of genes encoding pro-inflammatory cytokines (e.g. selenoprotein S) (4, 5). Previous studies have demonstrated that selenium supplementation has immunomodulatory effects in individuals with autoimmune (Hashimoto) thyroiditis in terms of lowering the concentrations of thyroid peroxidase antibodies, a finding which we recently confirmed in a large-scale randomised clinical trial (6, 7). It is thus speculated that selenium may have beneficial immunomodulatory effects that potentially can lead to better clinical outcomes (such as higher remission rates) in individuals with Graves’ hyperthyroidism.

Epidemiological studies have indeed linked an increased risk of Graves’ disease to low selenium status (5). Thus, one observational study showed higher serum selenium in individuals achieving remission than in individuals with relapse, and two other studies demonstrated lower selenium concentrations in individuals with newly diagnosed Graves’ disease compared with controls (8, 9, 10). Three trials showed faster restoration of biochemical euthyroidism with selenium as add-on to antithyroid drug (ATD) treatment compared with ATD alone (11, 12, 13). In contrast, two other trials found no effect of selenium on time to euthyroidism or rates of relapse (14, 15). In addition, a few trials have examined the effect of 6 months’ supplementation with selenium on thyroid eye disease (TED; also known as Graves’ orbitopathy), with predominantly favourable results. Limitations of these previous trials (16, 17, 18, 19, 20) included a low number of individuals, an unblinded or non-randomised study design, or a short period of follow-up. Although selenium supplementation is not recommended for Graves’ hyperthyroidism in the European Thyroid Association guideline (21), a 2018 survey found that 38% of European Thyroid Association members still recommend it for individuals with Graves’ disease without TED (22), which is only marginally lower than the 55% of a combination of European and American Thyroid Association members recommending selenium in cases of Graves’ disease with mild TED (23). Such a widespread use of selenium supplementation is striking, considering the fact that no sufficiently powered randomised trial has documented any clinically relevant effect of selenium supplementation in individuals with Graves’ hyperthyroidism.

We hypothesised that selenium supplementation has immunomodulatory effects that will improve clinical outcomes in individuals with Graves’ hyperthyroidism. To this end, we launched the Graves’ Selenium Supplementation (GRASS) trial to test whether 200 μg/day selenium supplementation as add-on to standard ATD treatment results in a higher disease remission rate (i.e. euthyroidism without further treatment) and improved QoL in individuals with Graves’ hyperthyroidism (24).

## Methods

### Study design

GRASS is a randomised, double-blinded, placebo-controlled, multi-centre trial in individuals with Graves’ hyperthyroidism. It investigated the effect of selenium supplementation compared with matching placebo in addition to ATD therapy. The trial design was pragmatic, i.e. a simple design mimicking routine clinical practice: few follow-up visits, routine settings, and routine personnel. The trial protocol has been published, and the trial was approved by the Ethics Committee in the Capital Region of Denmark (H-4-2012-026) (24). Participants were recruited from nine university hospitals (two in the Southern Region of Denmark and seven in the Capital Region of Denmark).

### Trial participants

Inclusion criteria were active Graves’ hyperthyroidism (TSH < 0.1 mIU/L and elevated levels of TRAb measured within the last 2 months) (first-time diagnosis or relapse), not yet receiving ATD or having received ATD for less than 2 months, and age ≥18 years. Exclusion criteria were major co-morbidities rendering the participants unlikely to continuously receive the trial intervention, previous treatment with radioactive iodine, ongoing ATD treatment for more than 2 months, ongoing treatment with immunomodulatory drugs, allergy to the components in the selenium and placebo pills, pregnancy or breastfeeding, daily intake of selenium supplementation >70 μg, and inability to read and understand Danish. Participants were recruited from the outpatient clinics at hospitals where they also received their usual treatment according to local and national clinical guidelines. Reported sex was based on the Danish Central Person Registry number (10-digit number), which corresponds to ‘sex assigned at birth’ unless changed by the participants. All participants provided written informed consent.

### Randomisation and masking

Eligible participants were randomly assigned (1:1) to selenium or matching placebo. Block randomisation was performed using randomly varying block sizes (four or six) and stratified according to disease status (incident or relapse) and clinical trial site. The allocation sequences were computer-generated by an external data scientist and incorporated into the PROgmatic trial management system (25). In addition, the allocation sequences were sent to the central hospital pharmacy in the Capital Region, where selenium and placebo tablets were packed in identical containers labelled with randomisation numbers. The central pharmacy also provided concealed opaque envelopes for each randomisation number in case of a need to break the code for any individual. Participants were enrolled by routine personnel, and the randomisation number for individual participants was provided by PROgmatic based on stratification variables. Blinding was maintained for personnel, investigators, and participants until all data analyses were completed.

### Procedures

The intervention was either 200 μg organic selenium as selenium-enriched yeast (consisting mainly of selenomethionine) or matching placebo tablets (i.e. non-selenium-enriched yeast). The appearance and taste of the tablets were identical, but the smell differed slightly between the selenium-enriched and non-selenium-enriched yeast. Selenium and placebo tablets were produced in Denmark by JemoPharm A/S (https://jemopharm.dk). The intervention was given in addition to standard treatment, and ATD withdrawal should be considered or attempted 18 months after randomisation at the latest. The intervention period for each participant was typically 24–30 months, as the intervention was taken until 12 months after ATD cessation (maximum trial duration: 30 months). Participants attended three trial visits (V1–3): at baseline, 18 months, and end of study. Clinical data were obtained from electronic medical records at all visits, as were participant-reported data and blood samples. Blood samples were initially stored at −80°C at the local trial centre and later transferred to Rigshospitalet, where they were stored at −80°C until batch analysis. The trial management system, PROgmatic, automatically collected electronic patient-reported outcomes at baseline, 6 and 12 weeks, 6, 12, 18, and 24 months, and at end of study. Participant compliance with the intervention during the trial was monitored by self-reported tablet intake at 6 and 12 weeks, and 6, 12, and 24 months. In addition, participants were asked about compliance at trial visits V2 (18 months) and V3 (end of study). Changes in serum selenium concentrations were used as a post hoc surrogate measure of trial adherence. We refer to the published trial protocol for a more detailed description of assessments (24).

### Outcomes

The primary outcome was the proportion of participants with the composite outcome of non-remission, defined as receiving ATD or having thyroid hyperfunction (TSH < 0.1 mIU/L) during the last 12 months of the intervention period or referral to ablative therapy (radioactive iodine or thyroid surgery). Referral to ablative therapy was based on routine clinical practice at the participating trial sites (i.e. it was not standardised), in keeping with the pragmatic trial design. The primary outcome was assessed centrally at Rigshospitalet, based on clinical data entered into PROgmatic at the local trial centres.

Secondary outcomes included thyroid-related QoL during the first year after randomisation and at the end of the intervention period (24–30 months), as measured by the ThyPRO composite QoL, hyperthyroid symptoms, and eye symptoms scale scores (26, 27). Other secondary outcomes were ATD treatment during the last 12 months of the intervention period; ablative therapy (thyroid surgery or radioactive iodine) during the intervention period; level of TRAb at 18 months and at the end of the intervention period (24–30 months); and number of participants with adverse or serious adverse reactions during the intervention period. At V2 and V3, participants were queried about adverse reactions. In addition, the electronic PROs contained specific questions about symptoms indicative of adverse reactions. An automated alert was sent to the local trial personnel in case participants reported such symptoms, and trial personnel subsequently contacted the participant for clarification.

### Statistical analysis

Sample size estimation was based on the primary outcome. The expected proportion of participants with non-remission was 50% in the placebo group. In our sample size calculation, we assumed that the proportion with non-remission was 37.5% in the selenium group. Thus, we needed 492 participants (246 in each intervention group) to be able to reject the null hypothesis with a power of 80% and a risk of type I error of 5%.

Baseline characteristics were summarised as frequency (%), mean (standard deviation (SD)), or median (interquartile range (IQR)). The following outcomes were analysed using logistic regression: proportion of participants with non-remission (primary); ATD treatment during the last 12 months of the intervention period (secondary); ablative therapy during the intervention period (secondary); incidence of TED (exploratory); and self-reported compliance with the intervention (exploratory). ThyPRO scale scores during the first year after randomisation were analysed using a baseline-adjusted mixed model with repeated measures. This method deals efficiently with missing data. ThyPRO scale scores at end of study were analysed by a general linear univariate model. Differences in ThyPRO scale scores between GRASS participants (at baseline and end of study) and a reference sample from the general population (28) were analysed using a general linear univariate model, adjusted for age and sex. Analyses of ThyPRO data were adjusted for multiple comparisons with the Benjamini–Hochberg method (29). Serum selenium as well as TRAb levels at 18 months and end of study were analysed by a baseline-adjusted mixed model with repeated measures. The number of participants with adverse or serious adverse reactions during the intervention period was analysed by a generalised linear model (Poisson distribution). Time to ATD withdrawal was analysed using a Cox proportional hazard rate model. Serum selenium concentrations of participants at visits V1–V3 were compared with those from a previously collected sample of the Danish general population (30) using an independent samples *t*-test.

All analyses were performed according to the intention-to-treat principle, i.e. participants were analysed according to their randomly allocated intervention assignment, regardless of their adherence to protocol. We refer to the published protocol for complete details of the statistical procedures, including sensitivity analyses (24). There was no data monitoring committee due to the low risk of the intervention. We conducted all analyses using SAS Enterprise Guide, version 8.4. The study was registered at ClinicalTrials.gov (NCT01611896).

## Results

**Figure 1:**
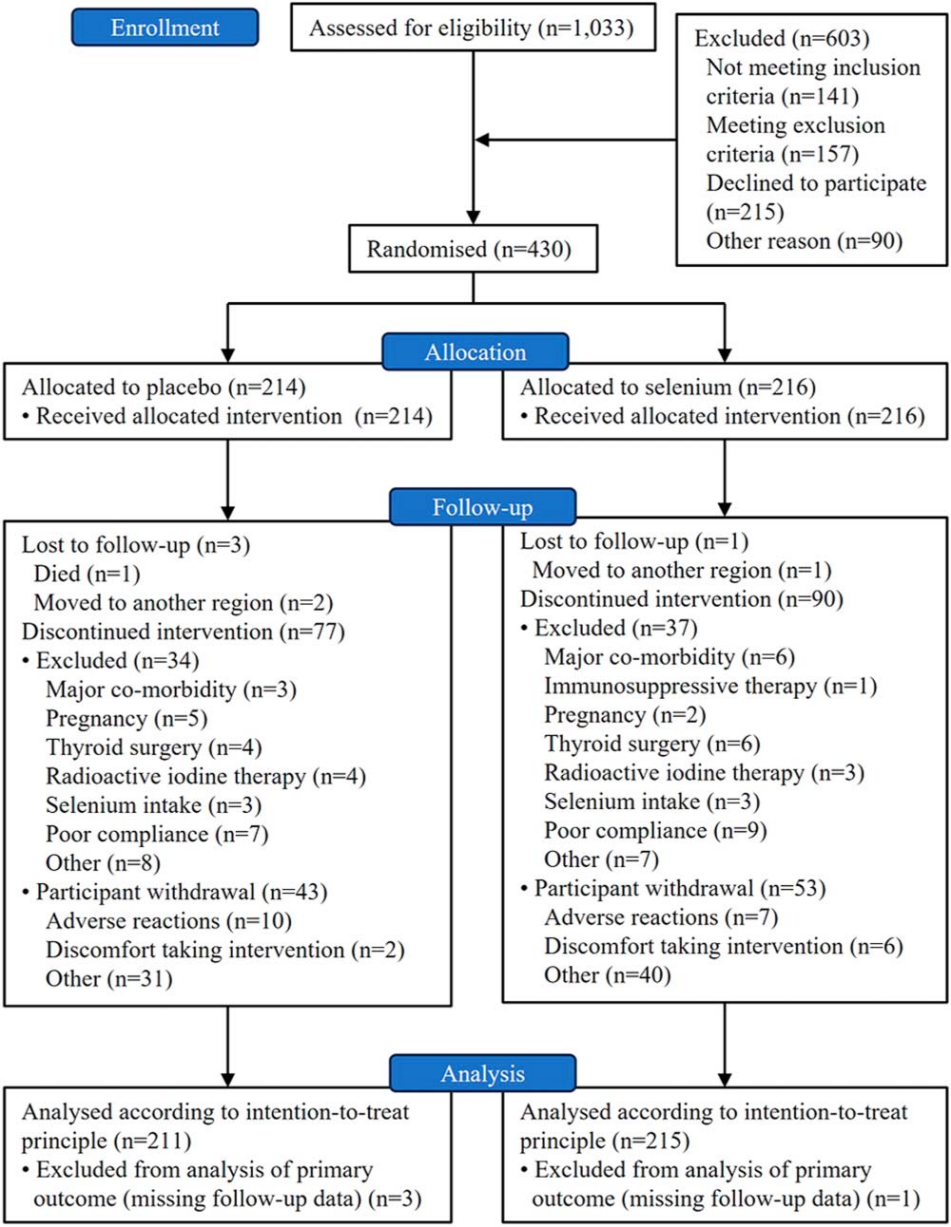
Flow diagram.

Between Dec 7th 2012 and Dec 3rd 2018, 430 participants with Graves’ hyperthyroidism were recruited from two Danish health care regions (Capital Region and Region of Southern Denmark) and randomly assigned to selenium supplementation (*n* = 214, 49.8%) or placebo (*n* = 216, 50.2%) (Fig. 1). The last participant completed the last visit on June 15th, 2021. Four participants were lost to follow-up, including one individual who died from causes not related to hyperthyroidism or the trial intervention. Three further participants moved to other health care regions. The four participants lost to follow-up were excluded from end-of-study analyses, but the collected data for these participants were included in all other analyses. The mean age of the randomly assigned participants was 49.0 years (SD: 13.1), 344 (80.0%) were female, 335 (77.9%) had incident hyperthyroidism, 95 (22.1%) had relapsed disease, and 29 (6.7%) were diagnosed with TED before the baseline visit. Eighty‐six (20.0%) participants were current smokers and 150 (35%) were previous smokers. There were no significant differences between the two intervention groups at baseline (Table 1).

**Table 1:** Baseline demographic and clinical characteristics of the participants. Data are presented as mean ± SD, *n* (%), or median (IQ1–IQ3).

	Placebo group (*n* = 214)	Selenium group (*n* = 216)
Age, years	49.6 ± 13.7	48.4 ± 12.5
Female sex	168 (78.5%)	176 (81.5%)
Smoking status		
Current smoker	43 (20.1%)	43 (19.9%)
Former smoker	77 (36.0%)	73 (33.8%)
Never smoker	80 (37.4%)	80 (37.0%)
Missing data	14 (6.5%)	20 (9.3%)
First-time GD or relapse of GD		
First-time	168 (78.5%)	167 (77.3%)
Relapse	46 (21.5%)	49 (22.7%)
Diagnosed TED	14 (6.5%)	15 (6.9%)
Laboratory values (serum)		
TSH, mIU/L*	0.01 (0.01–0.02)	0.01 (0.01–0.03)
FT4, pmol/L	19.5± 9.9	19.1± 9.2
TT3, nmol/L	2.6 ± 1.1	2.6± 1.1
T3-test	1.1 ± 0.2	1.1 ± 0.2
TRAb, IU/L	12.1 (±18.8)	14.0 (±26.5)^†^
TPOAb, kIU/L	533 (50–2,694)	404 (57–2,145)
TPOAb > 100 kIU/L	146 (68.2%)	143 (66.2%)
Selenium, μg/L	77.7 (±19.6)	82.4 (±38.3)^‡^

* TSH < 0.01 set equal to 0.01.

^†^ One outlier with very high TRAb (280 IU/L).

^‡^ One outlier with very high serum selenium (561 μg/L).

GD, Graves’ disease; FT4, free thyroxine; IQ, interquartile; TED, thyroid eye disease; TT3, total triiodothyronine; TPOAb, thyroid peroxidase antibodies; TRAb, TSH-receptor antibodies; TSH, thyroid-stimulating hormone.

Overall, 139 participants discontinued the trial intervention (baseline characteristics shown in Table 2). Participants who discontinued the trial intervention were younger than participants who completed it, 46.0 (SD: 14.1) versus 50.4 (SD: 12.3) years (*P* = 0.009), respectively. No other significant differences were observed between completers and non-completers (data not shown). Participants in the placebo group who discontinued the trial intervention due to adverse reactions (*n* = 10, Fig. 1), reported the following symptoms: headache (*n* = 2), gastrointestinal symptoms (*n* = 3), itchy skin (*n* = 1), lowered mood and decreased sex drive (*n* = 1), and unspecified adverse reactions (*n* = 3); and participants in the selenium group discontinuing the trial intervention (*n* = 7, Fig. 1) reported headache (*n* = 2), gastrointestinal symptoms (*n* = 3), and unspecified adverse reactions (*n* = 2). Participants discontinued the trial intervention due to adverse reactions after a mean duration of 9 (SD: 8) months and 6 (SD: 7) months in the placebo and selenium groups, respectively.

**Table 2:** Baseline demographic and clinical characteristics of the participants (*n* = 139) who discontinued the trial intervention. Data are presented as mean (±SD), *n* (%), or median (IQ1–IQ3).

	Placebo group (*n* = 60)	Selenium group (*n* = 79)	*P*-value
Age, years	48.1 ± 16.1	44.4 ± 12.3	0.13
Female sex	47 (78.3%)	69 (87.3%)	0.16
Smoking status			0.71
Current smoker	16 (26.7%)	15 (19.0%)	
Former smoker	18 (30.0%)	25 (31.6%)	
Never smoker	18 (30.0%)	29 (36.7%)	
Missing data	8 (13.3%)	10 (12.7%)	
First-time GD or relapse of GD			0.43
First-time	42 (70.0%)	60 (75.9%)	
Relapse	18 (30.0%)	19 (24.1%)	
Diagnosed TED	5 (8.3%)	7 (8.9%)	0.91
Laboratory values (serum)			
TSH, mIU/L*	0.01 (0.01–0.02)	0.01 (0.01–0.01)	0.16
FT4, pmol/L	20.2 ± 10.8	18.6 ± 9.1	0.36
TT3, nmol/L	2.7± 1.2	2.6 ± 1.2	0.66
T3-test	1.1 ± 0.2	1.1 ± 0.2	0.66
TRAb, IU/L	14.9 (±25.5)	18.3 (±38.3)^†^	0.41
TPOAb, kIU/L	426 (43–2,477)	907 (107–3,440)	0.29
Selenium, μg/L	75.8 (±22.7)	77.7 (±18.4)	0.59

* TSH < 0.01 set equal to 0.01.

^†^ One outlier with very high TRAb (280 IU/L).

GD, Graves’ disease; FT4, free thyroxine; IQ, interquartile; TED, thyroid eye disease; TT3, total triiodothyronine; TPOAb, thyroid peroxidase antibodies; TRAb, TSH-receptor antibodies; TSH, thyroid-stimulating hormone.

**Figure 2:**
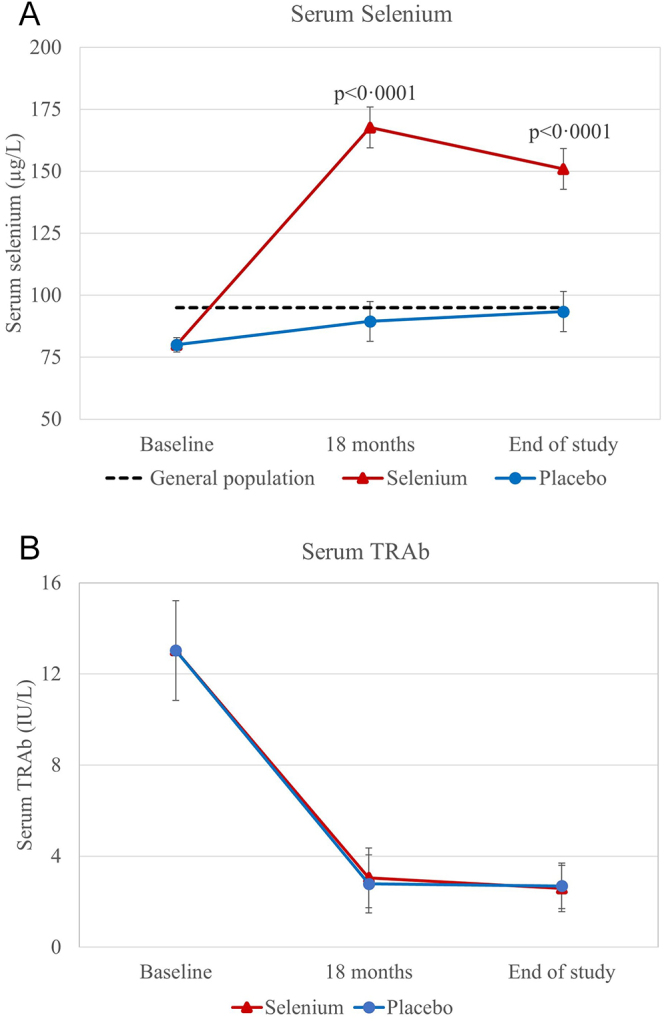
Serum selenium and serum TSH receptor antibodies (TRAb) during the intervention period. The data were analysed with a baseline-adjusted mixed-effect model with repeated measurements. (A) Serum selenium concentrations presented as predicted mean and 95% CI. Serum selenium was significantly higher at 18 months and at end of trial in the selenium group compared with the placebo group. The mean serum selenium concentration from a previously collected sample of the Danish general population (*n* = 401) is shown to facilitate the interpretation of patient data (30). (B) TRAb (secondary outcome) presented as predicted mean and 95% CI. No significant differences were observed between the groups.

Serum selenium measured at baseline, 18 months, and end of study was 82.4 (SD: 38.3) μg/L, 170.3 (SD: 71.2) μg/L, and 155.6 (SD: 71.7) μg/L in the selenium group, and 77.7 (SD: 19.6) μg/L, 88.4 (SD: 23.4) μg/L, and 92.5 (SD: 23.6) μg/L in the placebo group. In the selenium group, serum selenium increased from baseline to 18 months (mean difference (MD): 86.5 μg/L (95% CI: 74.7–98.3); *P* < 0.0001) and from baseline to end of study (MD: 71.8 μg/L (95% CI: 60.4–83.2); *P* < 0.0001). Serum selenium also increased, albeit slightly, in the placebo group from baseline to 18 months (MD: 10.3 μg/L (95% CI: 6.6–14.0); *P* < 0.0001) and from baseline to end of study (MD: 14.2 μg/L (95% CI: 10.3–18.1); *P* < 0.0001). Selenium levels were significantly higher in the selenium group at 18 months (MD: 78.2 μg/L (95% CI: 67.0–89.5); *P* < 0.0001) and at end of study (MD: 57.5 μg/L (95% CI: 46.3–68.8); *P* < 0.0001) compared with the placebo group (Fig. 2A). At baseline, participants in both intervention groups had significantly lower serum selenium concentrations than the Danish reference population. In the placebo group, selenium remained lower at 18 months but was comparable at study end, whereas in the selenium group, concentrations were significantly higher than the reference at both 18 months and study end.

**Figure 3:**
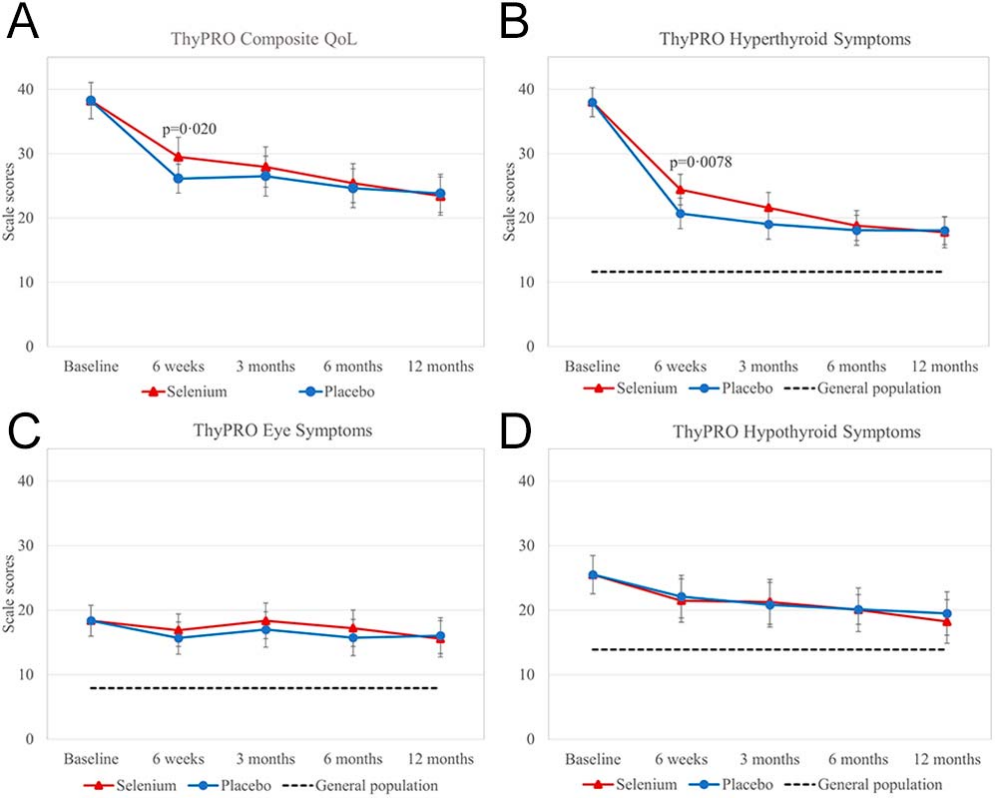
ThyPRO scale scores during the first 12 months of the intervention period. The data were analysed with a baseline-adjusted mixed-effect model with repeated measurements and presented as predicted mean and 95% CI. Reference scores from a previously collected general population (dotted line) sample are shown to facilitate the interpretation of patient scores (28). The composite QoL scale contains items asked with attribution to thyroid disease, which cannot be answered by respondents from the general population sample. (A) ThyPRO composite scale scores (secondary outcome). Significantly higher (worse) scores were seen at 6 weeks in the selenium group compared with the placebo group. This difference was not statistically significant after adjustment for multiple comparisons. (B) ThyPRO hyperthyroid symptoms scale scores (secondary outcome). Significantly higher (worse) scores were seen at 6 weeks in the selenium group compared with the placebo group. This difference was not statistically significant after adjustment for multiple comparisons. There were no significant differences for (C) the ThyPRO eye symptoms scale (secondary outcome) or (D) the ThyPRO hypothyroid symptoms scale (exploratory outcome).

There was no statistically significant difference in the proportion of participants with non-remission (primary outcome) when comparing the two randomisation arms (OR = 1.0 (95% CI: 0.7–1.5); *P* = 0.98) (Table 3). There was a trend towards a higher rate of remission in participants with newly diagnosed disease compared with those with relapsed disease (OR = 1.6 (95% CI: 1.0–2.5); *P* = 0.068). In addition, there were no statistically significant differences in the following secondary outcomes: ATD treatment within the last 12 months of the intervention period and ablative therapy during the intervention period (Table 3). Only one participant had suppressed TSH < 0.1 mIU/L during the last 12 months of the intervention period without receiving treatment with ATD. We observed no between-group differences in TRAb levels at 18 months (MD: 0.2 (95% CI: −1.5 to 2.0); *P* = 0.80) and at end of study (MD: −0.1 (95% CI: −1.5 to 1.3); *P* = 0.92) (Fig. 2B and Table 3). Furthermore, mixed-model analyses showed no group difference in the eye symptoms scale during the first 12 months of the intervention period, while participants in the selenium group had significantly higher (worse) scores on two scales compared with the placebo group: composite scale at 6 weeks (difference: 3.4 (95% CI: 0.5–6.2); *P* = 0.020) and hyperthyroid symptoms scale at 6 weeks (difference: 3.7 (95% CI: 1.0–6.4); *P* = 0.0078) (Fig. 3). In these three scales, we made 12 comparisons (between-group differences at 6 weeks, 3 months, 6 months, and 12 months). Hence, by adjusting for multiple comparisons with the Benjamini–Hochberg method, no significant differences existed at any time points in these three ThyPRO scales (29). Finally, we found no significant differences in scale scores between the intervention groups at end of study: composite (difference: −2.8 (95% CI: −7.4 to 1.7); *P* = 0.22), hyperthyroid symptoms (difference: −0.8 (95% CI: −4.3 to 2.6); *P* = 0.63), and eye symptoms (difference: 0.6 (95% CI: −3.2 to 4.4); *P* = 0.75). Participants with non-remission had significantly higher (worse) ThyPRO scores at the end of study on most scales as compared with participants in remission (data not shown). Adverse reactions to the trial intervention were reported for 24 participants in the selenium group and 21 in the placebo group (OR = 0.9 (95% CI: 0.6–1.5); *P* = 0.74) (Table 3). No participant experienced serious adverse reactions.

**Table 3:** Primary outcome and selected secondary and exploratory outcomes. Data are presented as *n* (%), predicted mean ± SE, or median (IQ1–IQ3). The four participants lost to follow-up were excluded from end-of-study analyses, but the data collected for these participants were included in all other analyses.

	Placebo group (*n* = 214)	Selenium group (*n* = 216)	*P*-values	OR or MD (CI)
Primary outcome				
Non-remission	114 (53.3%)	118 (54.6%)	0.98	1.0 (0.7–1.5)
Secondary outcomes				
ATD treatment within last 12 months	105 (49.1%)	104 (48.1%)	0.67	0.9 (0.6–1.4)
Ablative therapy	7 (3.3%)	13 (6.0%)	0.19	1.9 (0.7–4.8)
Level of TRAb, IU/L				
After 18 months	2.8 ± 0.7	3.0 ± 0.7	0.80	0.2 (−1.5 to 2.0)
End of intervention period	2.7 ± 0.5	2.6 ± 0.5	0.92	−0.1 (−1.5 to 1.3)
Adverse reactions	21 (9.8%)	24 (11.1%)	0.74	0.9 (0.6–1.5)
Serious adverse reactions	0	0	-	-
Exploratory outcomes				
Time to ATD withdrawal, days*	348 (252–453)	375 (299–455)	0.52	1.1 (0.8–1.5)^†^
Incidence of TED (CAS > 1)	5 (2.3%)	11 (5.1%)	0.14	2.2 (0.8–6.6)

* Only participants who were tapered out of ATD before 18 months and remained euthyroid, without further treatment, 1 year after cessation of ATD were included in this analysis.

^†^ Results reported as hazard ratio (CI).

ATD, anti-thyroid drugs; CAS, clinical activity score; IQ, interquartile; TED, thyroid eye disease; TRAb, TSH receptor antibodies.

**Figure 4:**
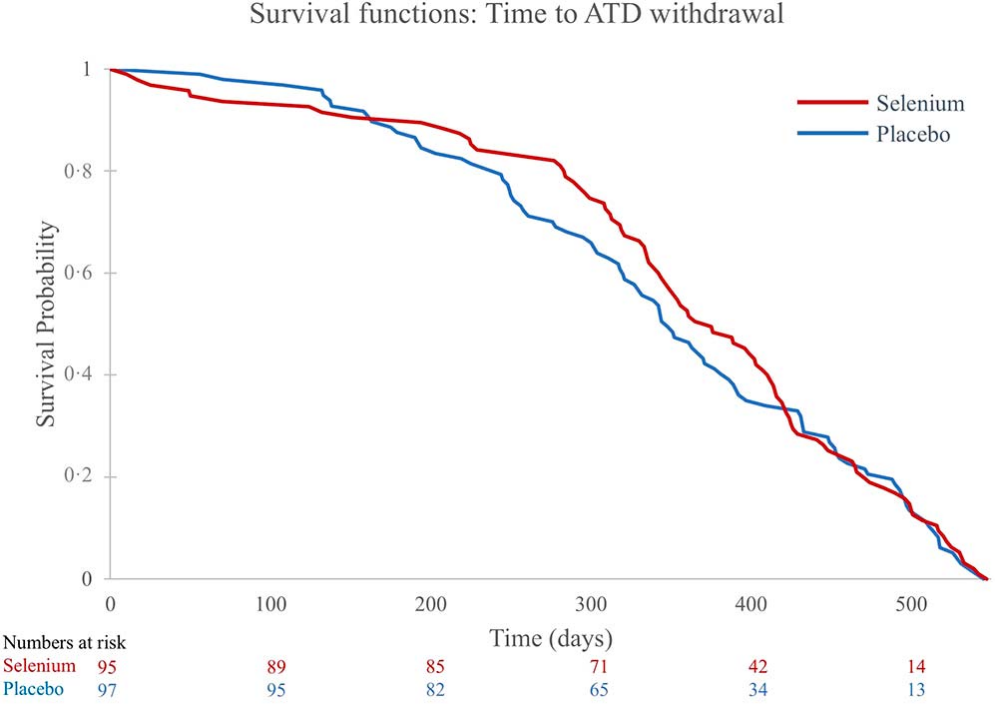
Graph of survival functions for time to ATD withdrawal displayed for the two intervention groups. Only participants who were tapered out of ATD before 18 months and remained euthyroid, without further treatment, 1 year after cessation of ATD were included in this analysis. The numbers at risk at each time point are shown immediately above the timeline. ATD, antithyroid drug.

Next, we analysed the pre-specified exploratory outcomes. Time to ATD withdrawal did not differ between the intervention groups (hazard ratio: 1.1 (95% CI: 0.8–1.5); *P* = 0.52) (Table 3 and Fig. 4). During the intervention period, 11 participants in the selenium group and five in the placebo group developed active TED (clinical activity score >1) (OR = 2.2 (95% CI: 0.8–6.6); *P* = 0.14) (Table 3). Three participants in the placebo group and two in the selenium group received intravenous glucocorticoid therapy for moderately to severe TED (OR = 1.3 (95% CI: 0.2–6.7); *P* = 0.79). There were no between-group differences on the hypothyroid symptoms scale during the first 12 months of the intervention (Fig. 3D), and no difference was observed at end of the study (difference: −1.7 (95% CI: −6.4 to 2.9); *P* = 0.46).

Sensitivity analyses did not significantly change the results reported above. There were no between-group differences in self-reported tablet intake. Subgroup analyses based on the level of increase in serum selenium from baseline to 18 months were performed to adjust for non-compliance: participants in the placebo group with the highest quartile of increase (cut-off: 21.4 μg/L), as well as participants from the selenium group with the lowest quartile of increase (cut-off: 43.7 μg/L), were excluded. These subgroup analyses did not show any significant differences in primary or secondary outcomes. An additional subgroup analysis limited to participants with baseline serum selenium concentration <80 μg/L (*n* = 123 per intervention group) also showed no significant differences in primary or secondary outcomes, although there was a trend towards a higher rate of non-remission in the selenium group (62 vs 55% in the placebo group). When further applying the cut-off values for non-compliance from above, there was an even stronger trend towards a higher rate of non-remission in the selenium group (70 vs 54% in the placebo group; OR = 1.4 (95% CI: 0.9–4.1); *P* = 0.08). Participants who went into remission had a mean baseline selenium of 83.7 μg/L compared with 76.7 μg/L for non-remission participants (difference: 6.9 μg/L (95% CI: 1.4–13); *P* = 0.025).

**Figure 5:**
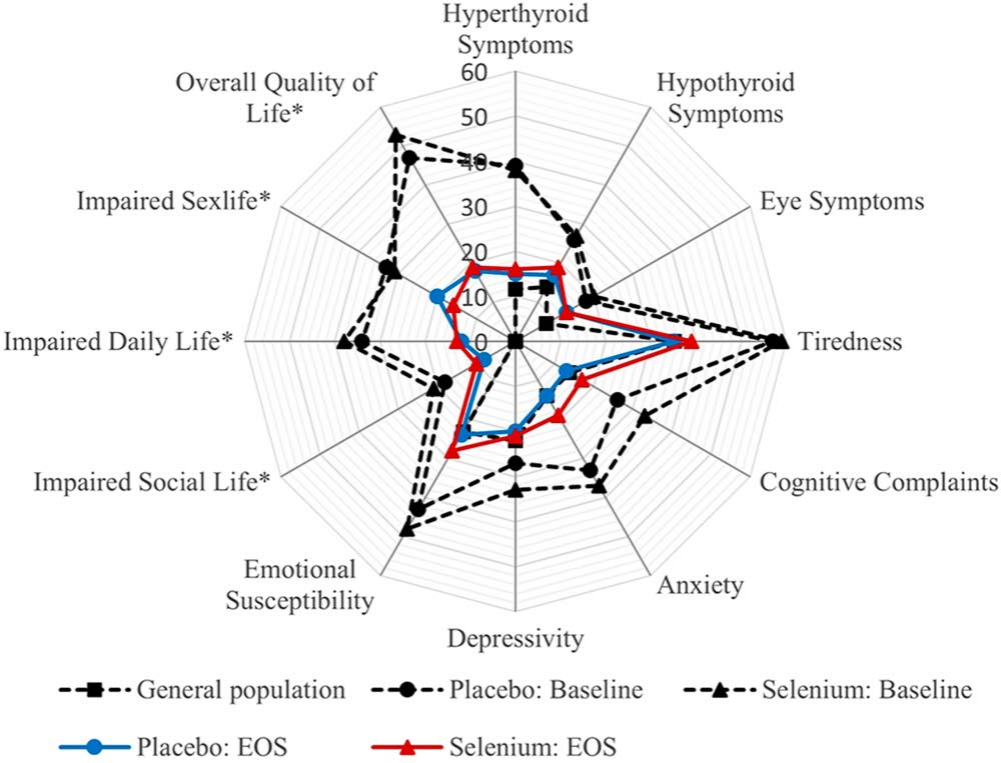
QoL at baseline and at end of study (EOS). Radar plot showing ThyPRO scale scores for the two intervention groups, as well as scores from a previously collected sample from the general population (*n* = 739) (28). The scales range from 0 to 100, with higher scores indicating worse health status. *Items in these scales are asked with attribution to thyroid disease and cannot be answered by respondents from the general population. See Table 4 for specific differences in scale scores.

Post hoc analyses examining differences in ThyPRO scale scores between GRASS participants and a previously collected reference sample from the general population are shown in Fig. 5 and Table 4 (28). At baseline, participants in both intervention groups scored significantly higher (worse) than the general population on all comparable scales, and the differences were larger than the minimal important change (MIC) for all scales except for depressivity scale scores in the placebo group (31). The participants’ QoL continued improving 6–12 months after trial inclusion (i.e. > 6–12 months after initiation of ATD) (Fig. 3). At end of study, participants in the placebo and selenium groups scored significantly higher (worse) on two and five scales, respectively; however, all differences were below the MIC for the ThyPRO scales (Table 4).

**Table 4:** Differences in ThyPRO scale scores between GRASS participants (at baseline and end of study) and a reference sample from the Danish general population. Data are presented as group differences (95% CI) and group-level Minimal Important Change (MIC) for the ThyPRO scales. Differences were analysed using a general linear univariate model, adjusted for age and sex. MICs were determined in a previous study (31).

ThyPRO scale	Baseline vs general population		End of study vs general population		Group-level MIC
	Placebo group	Selenium group	Placebo group	Selenium group	
Hyperthyroid symptoms	27.4 (25.0–30.0)*	25.9 (23.4–28.5)*	3.1 (0.6–5.7)*	4.0 (1.3–6.7)*	10.7
Hypothyroid symptoms	12.3 (9.6–14.9)*	13.4 (10.7–16.1)*	3.1 (0.0–6.2)	4.9 (1.6–8.1)*	6.3
Eye symptoms	10.2 (8.0–12.5)*	12.1 (9.8–14.3)*	5.5 (3.2–7.5)*	4.8 (2.4–7.2)*	6.3
Tired	23.0 (19.5–26.6)*	24.0 (20.4–27.6)*	0.9 (−3.3 to 5.0)	4.6 (0.2–9.0)	14.3
Cognition	12.1 (8.9–15.2)*	18.1 (15.0–21.2)*	−0.9 (−4.1 to 2.4)	3.1 (−0.4 to 6.5)	6.7
Anxiety	19.3 (15.9–22.7)*	23.2 (19.8–26.6)*	1.0 (−2.4 to 4.4)	5.3 (1.7–8.9)*	12.5
Depressivity	5.0 (1.8–8.1)*	10.6 (7.5–13.8)*	−2.2 (−5.7 to 1.3)	−1.2 (−4.9 to 2.5)	7.1
Emotional susceptibility	20.4 (17.0–23.8)*	24.2 (20.8–27.6)*	1.5 (−2.2 to 5.2)	5.5 (1.6–9.4)*	11.1

* *P* < 0.05 after adjustment for multiple comparisons with the Benjamini–Hochberg method.

## Discussion

The primary objective of the GRASS trial was to investigate whether selenium supplementation, as compared with placebo, as add-on to standard treatment with ATD leads to a higher rate of disease remission. Previous trials in Graves’ disease have shown contradictory results as to whether selenium supplementation leads to faster restoration of euthyroidism and higher remission rates (11, 12, 13, 15). However, these trials were all poorly powered, with 15 to 35 participants in each intervention group. In the present adequately designed and powered GRASS trial, selenium supplementation had no effect on remission rate or time to ATD withdrawal. Furthermore, there were no statistically significant differences between the intervention groups for any pre-specified secondary outcomes, including ATD during the last 12 months of the intervention period, thyroid ablation during the intervention period, TRAb levels at 18 months and end of study, or QoL. There were no differences in adverse reactions between groups, and no participants experienced serious adverse reactions. Overall, we assess that selenium supplementation was safe and well tolerated.

As many people in Denmark take multivitamins containing up to 55 μg selenium, participants were allowed to maintain this level of intake. Since Denmark is a borderline low selenium area, we expected that no patients would achieve very high levels of serum selenium, despite intake of multivitamins. Organic selenium, as selenium-enriched yeast, was chosen as the active intervention due to better bioavailability compared with inorganic selenium. The dose of 200 μg selenium was chosen to ensure that all patients in the active intervention group experienced a significant increase in serum selenium. The mean baseline serum selenium was 77.7 μg/L in the placebo group and 82.4 μg/L in the selenium group, which is lower than the 96.3 μg/L reported for 401 control participants in a Danish study conducted from 2004 to 2005 (30). This finding is in line with both a Danish and a Korean study, which demonstrated lower selenium concentrations in individuals with newly diagnosed Graves’ disease compared with control subjects (9, 10). Conversely, in a Chinese population study, low selenium status was not associated with an increased risk of Graves’ disease (32). While the issue of cause and effect is unsettled, these results suggest that Graves’ hyperthyroidism leads to lower selenium concentrations. Interestingly, we found that individuals who achieved remission had higher baseline selenium compared with those with persistent disease, which accords with previous studies (8, 9, 10). Thus, although we found no effect of selenium supplementation in individuals with active Graves’ hyperthyroidism, we cannot exclude that selenium supplementation may prevent the disease or hinder relapse in euthyroid predisposed individuals. This potential benefit of selenium supplementation should be addressed in future randomised studies.

A 2011 trial in individuals with TED demonstrated beneficial effects of 6 months of selenium supplementation compared with placebo in terms of improved QoL, reduced ocular involvement, and reduced disease progression (16). A few poorly powered trials have subsequently shown beneficial effects of a 6-month selenium course on orbital inflammation markers and other clinical outcomes in TED (17, 18, 19, 20). Participants in the present GRASS trial were not systematically examined by ophthalmologists, but were referred when deemed clinically relevant. The GRASS trial did not demonstrate any beneficial effects of selenium supplementation on TED incidence, eye symptoms, or need for glucocorticoid treatment or other intervention. However, TED at inclusion or during the trial was uncommon, and neither our design nor power allows conclusions about selenium supplementation and TED.

Ensuring adherence to study protocols is a well-recognised challenge in clinical trials. In the present trial, there were no between-group differences in self-reported tablet intake. Selenium concentrations increased markedly from baseline to the V2 and V3 visits in the selenium group. For participants recruited from late 2017 onwards, several follow-up visits coincided with the COVID-19 restrictions, leading to some visits being either skipped or delayed. Consequently, some participants completed their end-of-study visit after finishing the trial intervention, which may partly explain the lower selenium levels at end of study compared with 18 months in the selenium group. The selenium concentrations in the placebo group also increased from baseline to the V2 and V3 visits. The observed increase may reflect that some participants took selenium supplements, potentially attenuating the true between-group difference. However, selenium concentrations only rose to the background level of the Danish population. This rise may also reflect participants becoming euthyroid, which could reduce selenium turnover and thereby normalise serum selenium levels.

Strengths of the GRASS trial include its robust study design, its large sample size, and its long intervention period. We enrolled 430 participants and used an intervention and follow-up period of 24–30 months. In addition, the use of the extensively validated thyroid-related patient-reported outcome ThyPRO enhances the reliability and validity of the QoL assessments (26). Moreover, only four participants were lost to follow-up, and having access to electronic health records for all other participants, intention-to-treat analyses on complete clinical outcomes data could be carried out. Finally, the pragmatic trial design, mimicking daily clinical practice, maximises the clinical generalisability of our results.

The findings of this trial should be viewed in the context of its limitations. The trial was stopped before we reached the planned recruitment target of 492 participants because of funding constraints (24). Applying the assumptions of the power calculations used in the trial protocol, the power of the GRASS trial was reduced to 74.5%, while the minimal detectable effect for the primary outcome increased from 12.5 to 13.5%. We believe that the risk of a type II error is low, as no trends towards any beneficial effects of selenium were observed for any outcome measure. Nevertheless, the wide confidence intervals (Table 3) do not rule out clinically relevant effects – either beneficial or adverse – of selenium supplementation. In all, 139 participants discontinued the trial intervention, which we find acceptable given the long intervention period. Dropouts could potentially bias the trial results, but the characteristics of the participants who discontinued the intervention were similar in the selenium and placebo groups. In addition, the characteristics of the participants who completed and discontinued the intervention were similar, apart from a significantly lower age in the dropout group. The smell differed slightly between selenium-enriched (active intervention) and non-selenium-enriched (placebo) tablets, which potentially could lead to inadvertent unblinding, which again could affect the perceived benefits (e.g. affect the QoL scores). However, both tablet types had a strong yeast-like odour, which could easily be interpreted as active intervention. We recruited participants from nine university hospitals over a period of 6 years. The number of individuals with Graves’ disease referred to the trial sites during this period was clearly higher than the 1,033 individuals assessed for eligibility. Although we cannot exclude that individuals who were not assessed for eligibility differed significantly from the included individuals in terms of response to trial medication, we consider this risk to be low. Participants were only enrolled in Denmark, an area with a borderline low to adequate selenium status, which potentially limits generalisability to other geographical regions with different dietary selenium intake.

The GRASS trial is the first study that assesses QoL repeatedly over a long period (24–30 months). The QoL of the participants kept improving up to 12 months after initiation of ATD, which is valuable information for clinicians and patients. It is even more encouraging that the participants’ QoL was comparable to that of a general population sample at end of study, as all differences were less than the MIC for all ThyPRO scales. Nonetheless, there are still people with Graves’ disease experiencing long-term QoL impairments, but the mechanisms behind these are yet to be fully explored (3, 33). As recently demonstrated in another autoimmune thyroid disorder, Hashimoto’s thyroiditis, impaired QoL and persistent symptoms despite euthyroidism may be influenced by factors such as somatisation and type D personality (34). This association has not yet been explored in Graves’ disease. Routine use of QoL assessments in clinical practice has the potential to improve the clinical care of individuals with Graves’ hyperthyroidism by providing data on the performance of different treatments in ‘real-world’ conditions rather than under ideal or controlled circumstances (35). Routine QoL assessments could help identify the most optimal treatment strategies for effectively restoring QoL.

Current international guidelines recommend selenium supplementation for mild TED (21). However, there is no compelling evidence that selenium supplementation is beneficial for Graves’ hyperthyroidism *per se*. Accordingly, selenium supplementation – in the absence of orbitopathy – is not recommended in the European Thyroid Association guideline for the management of Graves’ hyperthyroidism (21). This lack of recommendation for selenium supplementation in Graves’ hyperthyroidism is supported by the results of the present trial. In view of the present findings, thyroid specialists’ widespread use of selenium in Graves’ disease, despite lack of support in thyroid guidelines, needs to be addressed in upcoming guidelines and when teaching specialists in endocrinology (22).

In conclusion, in this pragmatic, randomised, double-blinded, placebo-controlled trial of individuals with Graves’ hyperthyroidism, daily supplementation with 200 μg selenium did not significantly improve either the disease remission rate or thyroid-related QoL. The GRASS trial findings do not support routine selenium supplementation in Graves’ hyperthyroidism, as no evidence of clinically meaningful benefit was found.

## Declaration of interest

LS holds shares of selenOmed GmbH, a company involved in selenium status assessment. All other authors declare no competing interests.

## Funding

This research was funded by the Danish Agency for Science, Technology and Innovation (grant 271-09-0143), Danish Council for Independent Research (grant 09-066886), Ingeborg Brøste Svendsens Foundation as well as Agnes and Knut Mørks Foundation. UFR’s research salary was sponsored by a grant from Kirsten and Freddy Johansen’s Foundation. The funders of the trial had no role in trial design, data collection, data analysis, data interpretation, writing of the manuscript, or the decision to submit for publication.

## Author contribution statement

PKC, KHW, JBB, NK, PBM, RN, JG, BN, LH, SJB, UFR, ÅKR, and TW conceptualised and designed the study. PKC, JBB, JLF, and TW did the analysis. PKC drafted the manuscript. All authors contributed to data interpretation and writing of the final version of the manuscript. PKC, CBL, and TW accessed and verified the data. All authors had full access to the study data and were responsible for the decision to submit for publication.

## Data sharing

De-identified datasets generated and analysed during the current study are available from the corresponding author on reasonable request from qualified scientific and medical researchers. All requests will be reviewed by the GRASS steering committee, and data provided according to conditions laid by the GRASS steering committee. Furthermore, data transfer requires approval from the Danish Data Protection Agency and the Ethics Committee in the Capital Region of Denmark.
